# Relationship between hand size, volume of alcohol-based handrub and time needed to dry hands. An experimental laboratory-based study

**DOI:** 10.1186/2047-2994-4-S1-P303

**Published:** 2015-06-16

**Authors:** A Gayet-Ageron, F Bellissimo-Rodrigues, H Soule, Y Martin, D Pittet

**Affiliations:** 1 Infection Control Programme, University of Geneva Hospitals, Geneva, Switzerland

## Introduction

Quality of hand hygiene action is key to improve the effectiveness for health-care associated infection and antimicrobial resistance spread prevention. One of the parameters for quality is the volume of alcohol-based handrub (ABHR) used. The question around the possible need for customizing the volume of ABHR used to the hand size remains open.

## Objectives

To assess the relationship between the volume of ABHR used and the time needed to dry hands depending on hand size.

## Methods

Experimental study among a sample of healthy healthcare workers (HCW) trained to hand hygiene performance according to the WHO sequence. Six volumes of ABHR were tested (from 0.5mL to 3mL) and HCW were asked about their perception to obtain dry hands during the handrubbing sequence. Primary outcome was the acquisition of dry hands related to time of observation in seconds which was censored at 30 seconds. Due to clustered data, a shared frailty regression Cox model was performed.

## Results

Fifteen HCW were included (10 women; 66.7%). Four had small hand size (median surface hand 338.1 cm^2^), 6 medium (403.4 cm^2^) and 5 large (479.0 cm^2^). We collected 90 measurements from 15 HCW. After 30 seconds, only 35/90 hands (39%) were perceived as dry. Overall, median time described by HCW to obtain dry hands was 18 seconds (interquartile range 15-24). Acquisition of dry hands was significantly decreased for each additional volume of 0.5mL ABHR (hazard ratio, HR 0.14; 95%CI: 0.08-0.24, P<0.001) after adjustment on the hand size. Acquisition of dry hands was also significantly increased by hand size (P=0.03) with a higher HR for large compared to small hands (7.36; 95%CI: 1.64-33.06, P=0.009) and for medium compared to small hands (4.77; 95%CI: 1.10-20.56, P=0.036). There was no difference regarding the acquisition of dry hands between medium and large hands (P=0.486).

## Conclusion

Perception ofdry hands was significantly associated with the volume of ABHR used but also with the hand size. These results suggest the need for customizing hand hygiene duration with ABHR application according to HCW hand size but to keep also a sufficient volume of ABHR to ensure efficient antisepsis.

## Disclosure of interest

None declared.

